# Predictors of No-Show in Neurology Clinics

**DOI:** 10.3390/healthcare10040599

**Published:** 2022-03-22

**Authors:** Hisham Elkhider, Rohan Sharma, Sen Sheng, Jeff Thostenson, Nidhi Kapoor, Poornachand Veerapaneni, Suman Siddamreddy, Faisal Ibrahim, Sisira Yadala, Sanjeeva Onteddu, Krishna Nalleballe

**Affiliations:** 1 Department of Neurology, University of Arkansas for Medical Sciences, Little Rock, AR 72205, USA; nkapoor@uams.edu (N.K.); veerapaneni@live.com (P.V.); syadala@uams.edu (S.Y.); sronteddu@uams.edu (S.O.); knalleballe@uams.edu (K.N.); 2 Neurology Department Epilepsy Division, University of Pittsburgh Medical Center, Pittsburgh, PA 15213, USA; sen_sheng@outlook.com; 3 College of Public Health, University of Arkansas for Medical Sciences, Little Rock, AR 72205, USA; jdthostenson@uams.edu; 4 Department of Internal Medicine, Baptist Health, North Little Rock, AR 72117, USA; suman.siddam@gmail.com; 5 Department of Neurology, Cleveland Clinic Foundation, Cleveland, OH 44195, USA; ibrahif2@ccf.org

**Keywords:** neurology clinic, no-show, COVID-19

## Abstract

In this study, we aim to identify predictors of a no-show in neurology clinics at our institution. We conducted a retrospective review of neurology clinics from July 2013 through September 2018. We compared odds ratio of patients who missed appointments (no-show) to those who were present at appointments (show) in terms of age, lead-time, subspecialty, race, gender, quarter of the year, insurance type, and distance from hospital. There were 60,012 (84%) show and 11,166 (16%) no-show patients. With each day increase in lead time, odds of no-show increased by a factor of 1.0019 (*p* < 0.0001). Odds of no-show were higher in younger (*p* ≤ 0.0001, OR = 0.49) compared to older (age ≥ 60) patients and in women (*p* < 0.001, OR = 1.1352) compared to men. They were higher in Black/African American (*p* < 0.0001, OR = 1.4712) and lower in Asian (*p* = 0.03, OR = 0.6871) and American Indian/Alaskan Native (*p* = 0.055, OR = 0.6318) as compared to White/Caucasian. Patients with Medicare (*p* < 0.0001, OR = 1.5127) and Medicaid (*p* < 0.0001, OR = 1.3354) had higher odds of no-show compared to other insurance. Young age, female, Black/African American, long lead time to clinic appointments, Medicaid/Medicare insurance, and certain subspecialties (resident and stroke clinics) are associated with high odds of no show. Possible suggested interventions include better communication and flexible appointments for the high-risk groups as well as utilizing telemedicine.

## 1. Introduction

Many neurological disorders are projected to increase because of the aging population of the United States (US) [1]. For example, the incidence of stroke will raise by 20% in 2030, the incidence of dementia will double by 2050 and the prevalence of Parkinson’s disease will double by 2040 [1]. On the other hand, neurologists are in short supply while the demand for neurology services is growing. A study by the American Academy of Neurology in 2013 showed a projected shortage of neurologists to increase from 11% in 2013 to 19% by 2025 [2]. The average wait time to see a neurologist is more than 35 business days, which is longer than other specialties [2]. No-show in ambulatory neurology clinics does not only affect the efficiency and quality of workflow, but it also directly impacts the revenue and healthcare resources available to other patients. Moreover, clinic no show has significant implications on patients including delayed diagnosis and treatment, clinical worsening, and affects the management of medications that require close laboratory follow up such as anticonvulsant medications and immunotherapies [3].

The no-show rate in ambulatory clinics generally varies from 5 to 34% [4]. In one academic pediatric center, the yearly revenue loss from missed neurology appointments was $257,724, with a no-show rate of 26% [5]. The cost of missed appointments imposes a financial burden on hospitals. This effect may be compounded in nonprofit academic medical centers that see a high volume of patients who are uninsured or are on Medicaid [6].

Although several risk factors for no-show have been identified in prior studies, the evidence is limited in the field of adult neurology. In a survey sent to neurologists, no-show was the most bothersome patient behavior [7]. The neurology ambulatory clinic at the University of Arkansas for Medical Sciences is the largest clinic and the only academic neurology clinic in the state of Arkansas. This study aims to identify predictors of no-show in general neurology and neurology subspecialty clinics at our institution.

## 2. Materials and Methods

We conducted a retrospective review with data collected from clinic records from neurology clinics from July 2013 through September 2018, which included demographic data such as age, sex, race (self-reported), insurance status, and distance of patient’s residence from the hospital. Data on lead time (i.e., time between the scheduling date and the date of appointment); neurology subspecialty, and the date of appointment were also collected for analysis. Several categorical groups were defined, for analysis, within the study population. The groups included sex (male or female), age (younger (<60 years) or older (≥60 years)), quarter of the year in which the clinic appointment was scheduled (first, second, third, or fourth quarter), race (White/Caucasian, Black/African American, American Indian/Alaskan Native, Asian, Native Hawaiian/Other Pacific Islander, Other, or Un-known), insurance status (Medicare, Medicaid, or Other), lead time (less than 7 days, 7–14 days, 15–29 days, 30–59 days, or 60 days and more), and the distance of residence from the hospital (within 50 miles, greater than 50 miles). We compared the odds ratio (OR) of patients who missed the appointment (no-show) to those who made the appointment (show) in terms of the above-mentioned variables. Some had multiple clinic visits in our dataset, which required a repeated measure analysis. Data were analyzed using repeated measure multivariate logistic regression analysis to simultaneously compare the correlation of the variables with the no-show rate. We used SAS 9.4 software (SAS, Cary, NC, USA) for data analysis. The data were tested for interaction and variables with interaction were stratified to control for interaction. A *p* value of <0.05 was considered significant. The study is not human subjects research as determined by the University of Arkansas for Medical Sciences Institutional Review Board (number 239708).

## 3. Results

The total number of appointments in neurology clinics during the period from July 2013 through September 2018 was 71,178 with 60,012 (84%) show and 11,166 (16%) no-show patients. The demographics of our cohort are described in detail in Table 1.

With increase in lead time, the odds of no-show increased to 1.0019 times per day (*p* < 0.0001, 95% CI (1.0015, 1.0022)). When divided into subgroups the odds of no-show were significantly higher in the groups with longer lead time except between groups 15–29 days and 30–59 days. There was no difference in odds of no-show between new or follow-up appointments.

Looking at subspecialty clinics, the odds of no-show for movement disorders (*p* < 0.0001, OR = 0.4399, 95% CI (0.3872, 0.4998)), multiple sclerosis (*p* < 0.0001, OR = 0.6981, 95% CI (0.6262, 0.7782)), and neuromuscular (*p* < 0.0001, OR = 0.6258, 95%CI (0.5482, 0.7145)) clinics were significantly lower than general neurology clinics (Table 2). However, stroke (*p* < 0.0001, OR = 1.6212, 95% CI (1.3898, 1.8912)) and resident (*p* < 0.0001, OR = 1.3671, 95% CI (1.2171, 1.5357)) clinics had higher odds of no-show compared to general neurology clinics (Table 2). There was no difference in odds of no-show between epilepsy and general neurology clinics (Table 2). Patients with Medicare (*p* < 0.0001, OR = 1.5127, 95% CI (1.4371, 1.5922)) and Medicaid (*p* < 0.0001, OR = 1.3354, 95% CI (1.251, 1.4255)) had significantly higher odds of no-show compared to other forms of insurance (Table 2).

Women had a significantly higher no-show rate compared to men (*p* < 0.001, OR = 1.1352, 95% CI (1.0755, 1.1981)) (Table 2). The odds of no-show were lower in older patients (≥60 years) as compared to younger patients (<60 years) (*p* < 0.0001, OR = 0.49, 95% CI (0.47, 0.52)). The odds of no-show were higher amongst Black/African American patients as compared to White/Caucasian patients (*p* < 0.0001, OR = 1.4712, 95% CI (1.3853, 1.5625)), while the odds of no-show were lower in Asians (*p* = 0.03, OR = 0.6871, 95% CI (0.4405, 1.0717)) and American Indian/Alaskan Native (*p* = 0.055, OR = 0.6318, 95% CI (0.4188, 0.9532)) as compared to White/Caucasian patients. There was no difference between the odds of no-show between Native Hawaiian/Other Pacific Islanders and White/Caucasian patients (Table 2).

There was an interaction between groups by distance from the hospital and age. The distance groups were stratified by age to adjust for interaction. Older patients who lived close to the hospital (within 50 miles) had higher odds of no-show compared to older patients who lived farther away (more than 50 miles) (*p* ≤ 0.0001, OR = 1.2208, 95% CI (1.1038, 1.3502)). Young patients who lived close to the hospital (within 50 miles) had lower odds of no-show compared to young patients who lived farther away (more than 50 miles) (*p* < 0.0001, OR = 0.9115, 95% CI (0.8589, 0.9674)).

Patients had a higher tendency for no-show in the first (*p* = 0.003, OR = 1.104, 95% CI (1.046, 1.164)), second (*p* = 0.0002, OR = 1.104, 95% CI (1.047, 1.163)), and fourth (*p* = 0.01, OR = 1.072, 95% CI (1.013, 1.136)) quarters of the year compared to the third quarter (Table 2).

## 4. Discussion

Younger patients had significantly higher odds (19%, 8705 patients) of no-show compared to older people (10%, 2461 patients). More than half of the patients in neurology clinics were young (65%). Many of the older patients were retired and likely had no difficulties in finding the time to present to their clinic appointments compared to younger patients who worked and likely needed to schedule time off from work to go to the clinic. A systemic review of no-show in general practice showed that younger patients ranging in age from 17 to 40 years old were more likely to miss their appointments [8]. Another survey-based study from England demonstrated that the likelihood of no-show decreased with increasing age [9]. Many other studies showed similar findings [10,11,12].

There was a clear tendency for no-show in appointments with longer lead time. The likelihood of no-show increased with an increase in lead time. With 60 days or more lead time, 19% of the patients missed their appointment. The likelihood of no-show for clinic lead times of 30–59 days was 16%, of 15–29 days was 14%, of 7–14 days was 11%, and of 7 days or less was 8%. There appears to be a trend for an increase in no-show rates between the groups for 15–29 days and 30–59 days, but it was not statistically significant. Patients with 60 days or more of lead time represented 49% of the total number of patients in neurology clinics at our institute. Long lead time has been previously studied as a major factor for no-show. A study performed in an ophthalmology clinic showed an average no-show rate of 9.1% for resident clinics and 2.4% for faculty clinics with a lead time for the appointment of 2 weeks or less compared to no-show rates of 38.3% for resident clinics and 6.9% for faculty clinics for a lead time of 6 months [13]. Long lead time resulted in higher no-show rates in pediatrics clinics as well. A study showed a no-show rate of 23% for visits scheduled within 30 days compared to 47% for visits scheduled with a lead time of more than 30 days (*p* < 0.0001) [14]. Long delays for access to specialty referrals can increase Emergency Department costs and congestion [15]. As one study found, clinic visits with a lead time of more than 21 days after the scheduling date is a risk factor for Emergency Department visits [15]. Possible reasons for the increased odds of no show in patients with long lead time include resolution of the medical problem, seeking health care in a different institution, and forgetting about the clinic appointment.

Certain subspecialty clinics were associated with higher odds of no-show. Stroke (26%) and resident clinics (27%) had the highest no-show rates compared to other clinics. This was statistically significant when compared to the general neurology clinics. The no-show rates for movement disorder (7%), multiple sclerosis (10%), and neuromuscular clinics (12%) were lower. General neurology (21%) and epilepsy (20%) clinics had a relatively high no-show rate.

Patients with chronic and progressive diseases requiring continued care are more likely to present to appointments. A previous study had shown that patients with Parkinson’s disease had lower no-show rates compared to patients with other neurologic disorders [16]. The no-show odds were the lowest in movement disorder clinics in our study compared to other clinics, likely due to a large subset of patients with Parkinson’s disease. Patients in stroke clinics may have multiple comorbidities with persistent disabilities, which may act as barriers to clinic visits. Subspecialty has been shown to influence the no-show rates in orthopedic clinics as well [17]. As previously seen in an ophthalmology clinic study, our study also shows that the odds of no-show were higher for resident clinics compared to the faculty clinics [13].

In our cohort, 75% of the total number of patients were White/Caucasian and 23% were Black/African American. Black/African American patients had higher odds of no-show at 24% compared to White/Caucasian patients (13%). A study from the orthopedics clinic at the University of Alabama also showed that Black/African American patients were significantly more likely to miss their appointments [17]. Another study at a Veterans Affairs Primary Care Clinic showed Black/African American and non-White as predictors of no-show [12]. Wealth disparities between communities exist in the US [18]. Racial disparities also exist amongst Black/African American and White/Caucasian patients in health care [19,20]. It may be difficult for Black/African American patients to obtain time off work to go to a clinic visit that is scheduled during the weekdays. This is likely multifactorial with economics and level of education to state a few, which may have influenced the no-show rates in our study between these races. Opening clinics during weekends for high-risk populations and increasing the diversity of healthcare professionals may help mitigate theses disparities [21].

There are conflicting results in previous studies regarding sex influence on no-show rates. Most of the patients in our neurology clinic were women (63%) and they had significantly higher odds of no-show at 16% compared to 15% in men. There were higher odds of no-show in men in some studies [9,14,22,23], while another study from England showed a higher probability of no-show in women [10]. On the other hand, some studies showed no difference in the rate of no-show between men and women [18,24].

The rate of no-show was higher in the first, second, and fourth quarters of the year compared to the third quarter. It is unclear what may influence this, and further studies and patient no-show questionnaires may clarify this. When analyzed using insurance type, patients with Medicaid and Medicare had higher no-show rates of 24% and 15%, respectively, compared to 11% in patients with other forms of insurance. Patients with Medicaid represented 17% of the total number of patients. Medicaid insurance was a risk factor for no-show in prior studies for a pediatric neurology clinic [25] and a rhinology clinic [26]. In another study about patients in a cardiology clinic, patients with commercial insurance were more likely to keep their appointments than patients with Medicaid or Medicare, and uninsured patients [27]. The authors reported that this is an unexpected finding [27]. A co-payment is not required in their clinic and a suggested potential explanation is that this may not have been clearly communicated to all individuals [27]. More studies are required to identify the reasons behind the increased odds of no show among patients with Medicaid and Medicare insurance. Medicaid is a program to assist low-income patients with their medical expenses. Medicare is a medical insurance for people over 65, disabled people, and dialysis patients [28].

We had to stratify our data to adjust for the age when comparing groups on the basis of distance from the hospital. Older patients who lived close to the hospital (within 50 miles) had a higher rate of no-show compared to older patients who lived farther away (more than 50 miles). This may be because older patients living far away from the hospital had to plan for their clinic visit, while those who lived closer may have become complacent due to ease of access to the hospital. Young patients who lived close to the hospital (within 50 miles) had lower rates of no-show compared to young patients who lived farther away (more than 50 miles). Younger patients who live greater than 50 miles may have to take time off of work to travel to the clinic, while those who live closer may be able to fit the clinic visit into their daily schedule without taking a whole day off. In addition, areas farther from the hospital are more rural, and socioeconomic factors may have a role to play for younger patients living in rural areas. A study from a pediatric neurology clinic showed the distance from the clinic had an association with missed appointments [25].

There was no difference in the odds of no-show between new and follow up appointments. However, in a study conducted in another neurological outpatient clinic, new referrals have been associated with a significantly higher rate of a no-show [11].

### 4.1. Study Limitations

The effect of socioeconomic status, transport availability, recurrent no shows, appointment time (morning vs. afternoon), and patient disability were not studied. Another limitation is that this is a one-site study, which may result in selection bias. Only two age groups were studied: young (<60 years) and old (≥60 years). Sixty years was selected to differentiate young from old instead of 65 (the retirement age in most western countries). The WHO (World Health Organization) uses 60 as the cutoff point between young and old [29]. Other studies used 60 to differentiate young from old as well [30]. Assessment of the effect of reminders is another limitation in this study. Reminders either by a message or an automatic phone call were used in all appointments.

### 4.2. Suggested Interventions

Expanding clinic hours, shortening wait times by adding more clinic slots and providers, having dedicated personnel to explain the importance of continuity of care, improving communication, and sending reminders consistently to patients may help reduce the rate of no-show. These interventions may achieve their goals by primarily targeting the groups at risk of no show to their clinic appointments. This also requires a sufficient number of clinic support staff and schedulers. A quality improvement project to reduce the no-show rate in a pediatric neurology clinic in an academic center in Qatar showed improvement in no-show rate from 49% to 18% when they used interventions mainly addressing communication and appointment flexibility [31]. Calling patients who did not show up to their appointment or sending them a survey may provide more information regarding the reason of missing their appointment.

Another intervention that may improve the no-show rate is using telehealth, which allows clinic appointments to be conducted through real time interactive video in place of an in person visit. In one study, patients in teleneurology clinics consistently kept their appointments compared to face-to-face clinic visits despite the relatively short distances involved [32]. Another study showed that participants using telerehabilitation services completed significantly more appointments than participants attending in-person visits [33]. A study performed in pediatric neurology telemedicine clinics providing care to underserved patients showed a higher odds of appointment completion of telemedicine visits compared to in-person visits and concluded that telemedicine can serve as an equal adjunct to in-person clinic visits [34]. Telehealth has its limitations including concerns about privacy, inability to perform a complete physical examination, and limitations to personal interaction, but it has been demonstrated that telehealth helps in reducing the odds of clinic no show. Telehealth use has expanded in the setting of the COVID-19 pandemic and will likely continue even after the resolution of the pandemic.

## 5. Conclusions

In conclusion, we demonstrated that young age, female sex, Black/African American race, long distance for young patients, long lead time to clinic appointments, patients with Medicaid insurance, and certain subspecialties (resident and stroke clinics) are associated with higher odds for clinic no-show. On the other hand, the risk of no-show was low for movement disorder, multiple sclerosis, and neuromuscular clinics. To our knowledge, this study is the first on differences in the rate of no-show among neurology subspecialty clinics. Younger patients are more likely to miss their appointments, possibly because of work and other obligations. The association of sex with no-show was inconsistent in previous studies. Long lead time is an important risk factor for no-show and previous work has reached the same conclusion.

Suggested interventions include more communication and flexible appointments for high-risk groups. Moreover, telehealth utilization is expanding in the setting of the COVID-19 pandemic and will likely persist as a new normal after the resolution of the pandemic. Teleneurology is a promising intervention to reduce clinic no-show as observed in previous studies performed in pediatric neurology and rehabilitation clinics.

This study is one of a limited number of studies investigating factors correlating to attendance in adult neurology clinics. More work is needed for the evaluation of predictors of no-show and the effect of interventions on no-show. A prospective follow up study will be considered after applying the suggested interventions.

## Figures and Tables

**Table 1:** Study population characteristics (N  =  71,178).

Groups		Completed	No-Show	Total
Age	<60 years	37,405 (81%)	8705 (19%)	46,110 (64.78%)
	≥60 years	22,607 (90%)	2461 (10%)	25,068 (35.22%)
Lead time	<7 days	9259 (92%)	825 (8%)	10,084 (14.17%)
	7–14 days	6030 (88%)	818 (12%)	6848 (9.62%)
	15–29 days	6394 (86%)	1047 (14%)	7441 (10.45%)
	30–59 days	10,190 (84%)	1992 (16%)	12,182 (17.11%)
	≥60 days	28,139 (81%)	6484 (19%)	34,623 (48.64%)
Distance	<50 miles	25,204 (83%)	5091 (17%)	30,295 (42.56%)
	>50 miles	34,808 (85%)	6075 (15%)	40,883 (57.44%)
Quarter	first	14,924 (83%)	2988 (17%)	17,912 (25.17%)
	second	15,833 (85%)	2884 (15%)	18,717 (26.30%)
	third	16,798 (85%)	2960 (15%)	19,758 (27.76%)
	fourth	12,457 (84%)	2334 (16%)	14,791 (20.78%)
Subspecialty	General	2175 (80%)	542 (20%)	2717 (3.82%)
	Epilepsy	9675 (79%)	2634 (21%)	12,309 (17.29%)
	Headache	8838 (80%)	2204 (20%)	11,042 (15.51%)
	Movement Disorders	12,288 (93%)	966 (7%)	13,254 (18.62%)
	Multiple Sclerosis	13,544 (90%)	1447 (10%)	14,991 (21.06%)
	Neuro-Oncology-1	80 (85%)	14 (15%)	94 (0.13%)
	Neuro-oncology-2	18 (58%)	13 (42%)	31 (0.04%)
	Neuromuscular	5256 (88%)	726 (12%)	5982 (8.40%)
	Neuropsychology	90 (95%)	5 (5%)	95 (0.13%)
	Neurosurgery	144 (97%)	4 (3%)	148 (0.21%)
	Occupational Therapy	6 (67%)	3 (33%)	9 (0.01%)
	Resident	5115 (73%)	1899 (27%)	7014 (9.85%)
	Speech	1 (50%)	1 (50%)	2 (0.00%)
	Stroke	1195 (74%)	425 (26%)	1620 (2.28%)
	Urgent Clinic	1587 (85%)	283 (15%)	1870 (2.63%)
Gender	Women	37,809 (84%)	7292 (16%)	45,101 (63.36%)
	Men	22,198 (85%)	3871 (15%)	26,069 (36.63%)
	Unknown	5 (63%)	3 (38%)	8 (0.01%)
Race	American Indian/Alaskan Native	199 (88%)	27 (12%)	226 (0.32%)
	Asian	220 (90%)	24 (10%)	244 (0.34%)
	Black/African American	12,196 (76%)	3835 (24%)	16,031 (22.52%)
	Native Hawaiian/Other Pacific Islander	37 (80%)	9 (20%)	46 (0.06%)
	Other	713 (78%)	196 (22%)	909 (1.28%)
	Unknown *	387 (61%)	247 (39%)	634 (0.89%)
	White	46,260 (87%)	6828 (13%)	53,088 (74.58%)
Insurance	Medicaid	8984 (76%)	2854 (24%)	11,838 (16.63%)
	Medicare	20,022 (88%)	2659 (12%)	22,681 (31.87%)
	Other	31,006 (85%)	5653 (15%)	36,659 (51.50%)
Total		60,012 (84%)	11,166 (16%)	71,178 (100%)

* for use if patient refuses or fails to disclose.

**Table 2:** Comparison between groups based on age, sex, subspecialty clinic, race, yearly quarter, and insurance.

Parameter		Comparison Group	*p* Value	OR (95%CI)
Age		<60 years		
	≥60 years		<0.0001	0.4873 (0.4569, 0.5196)
Sex		Male		
	Female		<0.001	1.1352 (1.0755, 1.1981)
Subspecialty		General Neurology		
	Movement Disorder		<0.0001	0.4399 (0.3872, 0.4998)
	Multiple Sclerosis		<0.0001	0.6981 (0.6262, 0.7782)
	Neuromuscular		<0.0001	0.6258 (0.5482, 0.7145)
	Stroke		<0.0001	1.6212 (1.3898, 1.8912)
	Resident		<0.0001	1.3671 (1.2171, 1.5357)
	Epilepsy		0.31	
Race		White/Caucasian		
	Asian		0.03	0.6871 (0.4405, 1.0717)
	Black/African American		<0.0001	1.4712 (1.3853, 1.5625)
	American Indian/Alaskan Native		0.055	0.6318 (0.4188, 0.9532
Yearly quarter		Third		
	First		0.0003	1.104 (1.046, 1.164)
	Second		0.0002	1.104 (1.047, 1.163)
	Fourth		0.01	1.072 (1.013, 1.136)
Insurance		Other		
	Medicare		<0.0001	1.5127 (1.4371, 1.5922)
	Medicaid		<0.0001	1.3354 (1.251, 1.4255)

## Data Availability

Individual de-identified subjects data will be shared at the request of other investigators.
